# Low coverage of HPV vaccination in the national immunization programme in Brazil: Parental vaccine refusal or barriers in health-service based vaccine delivery?

**DOI:** 10.1371/journal.pone.0206726

**Published:** 2018-11-12

**Authors:** William Mendes Lobão, Fernanda Gross Duarte, Jordan Danielle Burns, Carlos Antonio de Souza Teles Santos, Maria Conceição Chagas de Almeida, Arthur Reingold, Edson Duarte Moreira

**Affiliations:** 1 Gonçalo Moniz Institute, Oswaldo Cruz Foundation, Salvador, Brazil; 2 School of Nursing, State University of Bahia, Salvador, Brazil; 3 School of Public Health, Berkeley, CA, United States of America; 4 Rollins School of Public Health, Emory University, GA, United States of America; 5 Charitable Works Foundation of Sister Dulce, Salvador, Brazil; University of Campania, ITALY

## Abstract

**Background:**

The World Health Organization has recommended the introduction of HPV vaccines into national immunization programme (NIP), but vaccination coverage remains low worldwide. We assessed the coverage and the parental acceptance of female and male HPV vaccination in Brazil after its introduction into the NIP.

**Methods:**

We conducted a random-digit-dial survey of parents in seven major Brazilian cities from July-2015 to October-2016. A knowledge, attitude and practices questionnaire was developed and validated by expert analysis, semantic analysis, and pre-testing.

**Results:**

826 out of 2,324 (35.5%) eligible parents completed the interview. Parental acceptance of the HPV vaccine for daughters and sons 18 years of age or less was high (92% and 86%, respectively). Parents refusing vaccination were less likely to know that: HPV is sexually transmitted and causes genital warts, HPV vaccination is more beneficial before sexual debut, and HPV vaccine reactions are minor, and they were more likely to believe HPV vaccination can cause severe adverse events. Parents accepting HPV vaccine for daughters but not forsons were more likely to ignore that the vaccine is recommended for boys. Attitudes associated with HPV vaccine acceptance included: general belief in vaccines, trust in the NIP and in the HPV vaccine efficacy. Among girls eligible for HPV vaccination through the NIP, 58.4% had received a two-dose scheme and 71.1% at least one dose. “No vaccination/missed vaccination at school” was the most common reason for missed HPV vaccination in theNIP.

**Conclusions:**

One year after introduction in the NIP, most parents surveyed in Brazil accepted HPV vaccination for their daughters and sons. Low coverage in the NIP seemed to be due to challenges in adolescent vaccine delivery and HPV vaccination barriers at health-care centers, rather than to vaccine refusal.

## Introduction

Human papillomavirus (HPV) is responsible for nearly all cases of cervical and anal cancers, approximately 70% of the cancers affecting the vagina, vulva, and oropharynx, and 60% of penile cancers[1]. In Brazil, according to recent estimates from the International Agency for Research on Cancer, 8,414 women die from cervical cancer and 18,503 new cases are diagnosed annually, ranking as the 2^nd^ most frequent cancer among women between 15 and 44 years of age in Brazil[2].

There are currently three highly effective and safe licensed vaccines against HPV. The World Health Organization has recommended the introduction of HPV vaccines into immunization programme for children and young adults[3]. Nevertheless, HPV vaccination coverage has been disappointingly low worldwide, and only 1.4% of all eligible females have received a full-course of HPV vaccination[4]. Furthermore, there is inequity in access to HPV vaccines, in high income regions 33.6% of females aged 10–20 years have received the full course of HPV vaccine, compared with only 2.7% in lower income regions[4]. Hence, populations of countries carrying most of the burden of HPV-related diseases worldwide have the least access to the vaccines[5].

The quadrivalent HPV vaccine was introduced into the National Immunization Programme (NIP) in Brazil in 2014, targeting girls 9 to 13 year of age. Initially, the vaccination was school-based and the schedule included two doses 6 months apart followed by a third dose 60 months later. Later in the first year, a reduced two-dose schedule was adopted and the vaccination delivery changed to a health-clinic based strategy. The programme was extended to boys 11 to 13 years old in 2017. According to NIP data for 2014 to 2017, the cumulative vaccine coverage for the two-dose course in girls was 45.1%, and 72.4% of the targeted female population received at least one dose[6]. The coverage for at least one dose in boys was disappointingly low at 20.2%[6]. It has been argued that the low uptake of HPV vaccine in Brazil may be due to fear of adverse reactions (following media reports of neurological symptoms in clusters of girls in Brazil), parental vaccine hesitancy, and/or logistical challenges to vaccinating adolescents at health-care centers[7]. However, there is no data available to indicate which one of these reasons (or whether combination of them) is to blame for that. Previous studies in other countries show vaccine delays and low uptake related to vaccine hesitancy and barriers in access to vaccines[8–12].

The aim of this study was to assess coverage and parental acceptance of the HPV vaccine for adolescent daughters and sons in Brazil after its introduction into the NIP. In addition, we sought to determine factors associated with parental intentions for female and male HPV vaccination.

## Methods

We conducted a cross-sectional study in seven Brazilian cities (Belém, Belo Horizonte, Brasília, Porto Alegre, Rio de Janeiro, São Paulo, and Salvador). All five Brazilian regions were included. The interviews were conducted by telephone from July/2015 to October/2016. All participants gave verbal consent prior to commencing the interviews. This study was approved by the Ethics Committee of the Gonçalo Moniz Research Center—CPqGM / FIOCRUZ-BA(Protocol CAAE: 31234914.6.0000.0040; Approval number: 738.720).

### Study sample

Our sample size was estimated at 801, based on an estimate of parental acceptance of the HPV vaccine of 75%, the population size of the 7 cities 29,298,142, a 95% confidence interval and 3% margin of error.

Participants were selected by random-digit-dialing. Briefly, we sampled from a computer-generated list of all published telephone numbers in each city selected. Parents in households with children aged 18 years or younger were identified and invited to participate. When an eligible parent was not immediately available, a follow-up appointment was scheduled. If the selected person was unwilling to participate, no substitution was made in that household. Up to 10 callbacks were made to repeated no-answers, busy phone numbers, and answering machines.

### Data collection

All interviewers were trained and certified before study enrollment. A round of pilot testing was conducted prior to data collection to assess and improve question wording. We developed and validated aknowledge, attitude and practices (KAP)[13] questionnaire (S1 Questionnaire and S2 Questionnaire). The questionnaire included 79 items grouped in six categories: socio-demographic data, knowledge, attitudes, health practices, and HPV vaccination. The validity of the questionnaire was assessed by expert analysis (five researchers with substantial experience in field survey and epidemiological studies), semantic analysis, and pre-testing. Prior to data collection, a round of pilot testing was conducted to assess and improve question wording. Thirty pilot interviews were recorded, and then three reviewers listened to each interview of this pilot test, some survey questions were reworded or eliminated, and additional training was provided to interviewers.The expert and the semantic analysis, while considered part of the construction of the questionnaire, were steps in the initial validation of content.

### Statistical analysis

Characteristics of the study population and parental acceptance of the HPV vaccine were presented as numbers and/or percentages of participants, and stratified by parental acceptance of HPV vaccination. The statistical significance (two-tailed p<0.05) was assessed by the Chi-square (χ^2^) or Fisher’s exact testfor categorical variables. For each assessment about HPV or HPV vaccine, parents’ knowledge was arbitrarily classified as “adequate” if 70% or more of the answers were correct, otherwise, knowledge was considered “inadequate”.All statistical analyses were performed using Stata Statistical Software (College Station, TX: Stata Corp LP). The dataset is available (S1 Dataset)

## Results

Out of 2,324 eligible parents, 826 completed the interview for a response rate of 35.5%. The majority were women (85%), with a mean age of 43.8 years (range 18 to 82). At the time of the study interview, 37%had at least one daughter/son in the age range for HPV vaccination (9 to 14years)(Table 1).

**Table 1:** Socio-demographic characteristics of 826 parents in Brazil, 2015–2016.

	n	%
Sex (n = 826)		
Female	704	85
Male	122	15
Age (n = 787)		
< 20 years	21	3
20–29 years	94	12
30–39 years	235	30
40–49 years	222	28
≥ 50 years	215	27
Race/Ethnicity (n = 786)		
White	346	44
Mixed	324	41
Black	106	14
Asian	8	1
Indigenous	2	0,3
Marital Status (n = 790)		
Married	522	66
Single	156	20
Divorced or separated	75	10
Widowed	37	5
Religion (n = 791)		
Catholic	436	55
Evangelical / Protestant	237	30
Spiritist	48	6
Other	16	2
No religion	54	7
Current Occupation (n = 788)		
Employed	462	59
Homemaker	169	21
Retired	69	9
Unemployed	55	7
Student	33	4
Educational Attainment (n = 788)		
No formal education	11	1
Primary school or less	145	18
High school (graduate or some)	367	47
College (graduate or some)	265	34
Other Characteristics (n = 790)		
Has a daughter in the age range for HPV vaccination	291	37
Has private health insurance	435	55
City (n = 826)		
Belém	115	14
Belo Horizonte	117	14
Brasília	117	14
Porto Alegre	116	14
Rio de Janeiro	115	14
Salvador	129	16
São Paulo	117	14

### Parental Acceptance of HPV vaccine

**Fig 1:**
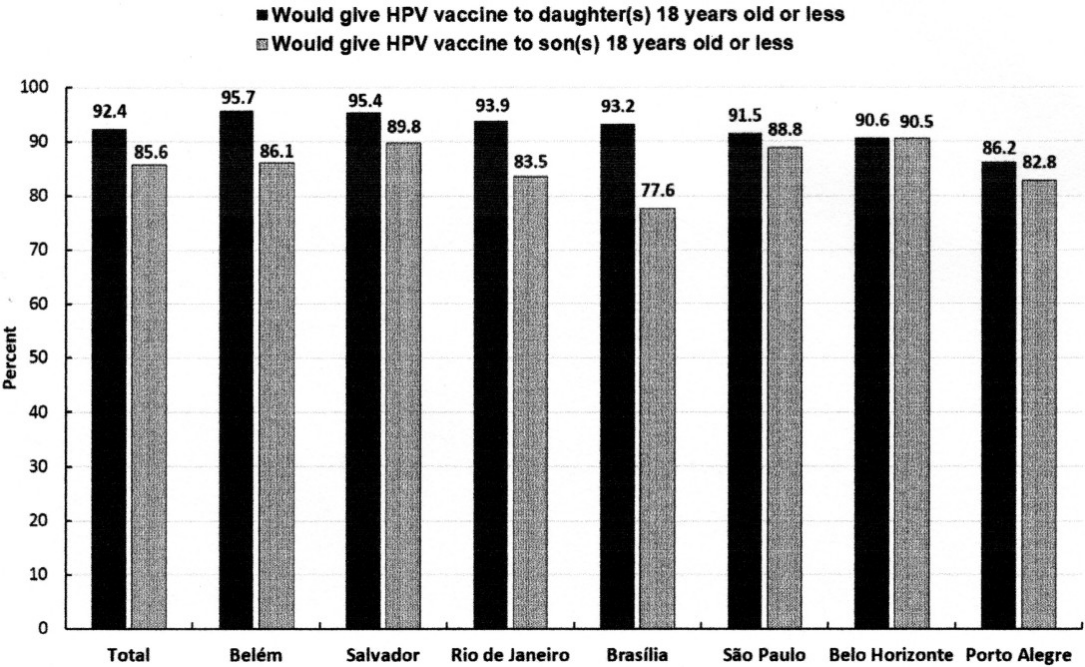
Parental acceptance of Human Papillomavirus (HPV) vaccination for daughter(s) or son(s)age18 years or less (n = 826), Brazil, 2015 to 2016.

The parental acceptance of HPV vaccine for daughters or sons less than 18 years of age was high (92% and 86%, respectively), and did not differ significantly in the different cities surveyed (Fig 1). Vaccine acceptance for daughters was comparable among mothers and fathers, 92.8% vs. 90.2% (p = 0.319), respectively;the same was also true for sons (mothers:85.9%vs. fathers:84.0%; p = 0.592).

### Knowledge, attitudes and health practices about HPVand the HPV vaccine

Parents’ knowledge about HPV and HPV vaccine was considered adequate for 10 of 21 items assessed (47.6%). Less than one third of parents (30%) knew that there was a vaccine to prevent genital warts, and 37% acknowledged that condoms are not fully protective against HPV infection (Table 2). Parents accepting HPV vaccination were more likely than parents refusing the vaccine to know that: HPV is sexually transmitted, HPV can cause genital warts, HPV vaccine is more beneficial when given before sexual debut, and HPV vaccine most common reactions are minor. Parents accepting HPV vaccine for their daughters but refusing it for their sons were less likely than either parent who accept it for all children or those who refused the vaccine to know that HPV vaccination is recommended for boys (Table 2).

**Table 2:** Knowledge, attitudes and practices about human papillomavirus (HPV) and the HPV vaccine according to parental acceptance of HPV vaccination, Brazil, 2015–2016.

	Total	Parental acceptance of HPV vaccination ^a^			
		Accept for both daughters and sons	Accept for daughters (but not for sons)	Refuse vaccination	P-value
	(n = 807)^b^	(n = 689)	(n = 69)	(n = 49)	
**Knowledge items** ^c^					
Transmission of HPV					
HPV is transmitted by sexual contact (T)	92	94	90	82	**0.007**
HPV is spread by airborne transmission (F)	92	92	88	88	0.347
Even without symptoms, someone can transmit HPV (T)	83	84	83	80	0.762
HPV is transmitted through use of public bathrooms / pools / showers (F)	66	65	74	67	0.357
Condom use fully protects against HPV (F)	37	36	38	43	0.636
Epidemiology and clinical aspects of HPV and cervical cancer					
HPV can cause cervical cancer (T)	86	86	90	79	0.286
Cervical cancer is NOT a common cause of cancer death among women (F)	75	76	69	67	0.176
HPV does not cause cancer in men (F)	75	76	73	69	0.511
HPV can cause genital warts (T)	69	71	61	58	0.055
Men cannot catch HPV (F)	66	66	67	61	0.771
HPV can be cured with antibiotics (F)	62	61	59	74	0.219
Someone with HPV usually has symptoms (F)	58	57	65	67	0.168
HPV is a very common virus (T)	55	56	48	61	0.316
Primary prevention of HPV / Cervical cancer					
A vaccine against HPV already exists (T)	89	89	90	82	0.279
The HPV vaccine works better when it is given before the start of sexual activity (T)	87	89	87	67	**<0.001**
The HPV vaccine is not for boys (F)	71	72	52	79	**0.001**
The most common reactions from the HPV vaccine are minor, such as pain and discomfort at the injection site (T)	65	66	71	38	**<0.001**
There is a vaccine against cervical cancer (V)	65	65	63	71	0.637
There is no vaccine against genital warts (F)	30	30	28	23	0.537
Secondary prevention of HPV / Cervical cancer					
GIRLS that receive the HPV vaccine do not need to have preventive exams (F)	82	82	76	83	0.425
If a preventive exam/Pap smear is normal, then a woman does not have HPV (F)	64	63	61	69	0.666
**Attitudes items** ^d^					
Confidence in vaccines (efficacy/safety)					
I generally believe in vaccines	96	97	93	84	**<0.001**
I trust the National Immunization Programme	94	96	91	71	**<0.001**
If the HPV vaccine worked for any age, I would get it	92	94	93	55	**<0.001**
The HPV vaccine is efficacious/ it works	83	85	84	43	**<0.001**
I don’t think the HPV vaccine is safe/ I think it can cause severe reactions	21	18	23	67	**<0.001**
Perception of Risk					
I would give my child a vaccine against a sexually transmitted infection	72	74	71	58	0.066
I think my DAUGHTER is at risk/has a chance of getting HPV	71	72	71	57	0.102
I think my SON is at risk/has a chance of getting HPV	68	72	36	54	**<0.001**
GIRLS between 9 and 13 years are too young to get the vaccine	22	18	23	67	**<0.001**
Getting the HPV vaccine can cause GIRLS to become sexually active much earlier	15	14	16	35	**<0.001**
**Health Practices and Medical History** ^e^					
Have had a cervical cancer screening Pap test at least once before ^f^	92	93	85	93	0.109
Have had a cervical cancer screening Pap test in the past three years ^f^	83	84	75	90	0.146
Have had the Diphtheria, Tetanus, and Pertussis vaccine	69	70	63	68	0.52
Have had the Hepatitis B vaccine	66	66	63	68	0.803
Know other parents who had their children vaccinated with HPV vaccine	59	61	58	40	**0.022**

^a^All parents had at least one child age <18 years old (boy or girl). Thus, the context of having a child for whom they decide about vaccination is always real, but the parental responses regarding either girls or boys can be hypothetical.

^b^The total (n = 807) represents participants responding to these items.

^c^Percentage of parents with correct answers regarding the statements (True or false).

^d^Percentage of parents who agreed with the statement.

^e^Percentage of parents responding affirmatively.

^f^Data refer only to female participants.

Parental attitudes significantly associated with HPV vaccine acceptance included: beliefs in vaccines in general, trust in the NIP, belief in the efficacy of HPV vaccine, and willingness to receive the HPV vaccine if recommended. In contrast, parents were more likely to refuse HPV vaccination if they believed that: HPV vaccine is not safe or can cause severe reactions, girls age 9 to 13 years are too young to get HPV vaccine, and HPV vaccination can cause girls to become sexually active earlier. Parents refusing HPV vaccine for boys were less likely to perceive their sons as being at risk of getting HPV infection (Table 2).

Knowing other parents who had their children vaccinated against HPV was associated with accepting HPV vaccination. Most mothers in our survey have had a cervical cancer-screening test performed at least once in their lifetime (92%) or in the past three years (83%). A full course of diphtheria, tetanus and pertussis vaccine or hepatitis B vaccine was reported by 69% and 66% of the study participants, respectively (Table 2). None of these health practices was associated with acceptance of HPV vaccination.

### Reasons for acceptance or refusal of HPV vaccination

The reasons for parental acceptance or refusal of human papillomavirus (HPV) vaccination, using open-ended questions to elicit spontaneous responses, are presented in Table 3. The most common motive for accepting vaccination for daughters and sons was that “vaccination is good/important” cited as the primary reason by 90% of the parents, and as one of the reasons by 96% of them. Cancer prevention was the second most common reason cited by only 7% of parents as the primary reason, and as one of them by 10%. Parents accepting HPV vaccination for girls but refusing it for boys cited cancer prevention more often, either as the primary reason (15%) or as one of the reasons (22%).

**Table 3:** Frequency distribution (%) of the reasons for acceptance or refusal of human papillomavirus (HPV) vaccination reported by parents, using open-ended questions to elicit spontaneous responses,n = 804, Brazil, 2015–2016.

	Parents accepting vaccination of daughters and sons (n = 687)		Parents accepting vaccination of daughters but not of sons (n = 68)		Parents refusing vaccination (n = 49)	
	Reported as the primary reason	Reported as one of the reasons	Reported as the primary reason	Reported as one of the reasons	Reported as the primary reason	Reported as one of the reasons
**Reasons for acceptance of HPV vaccination**						
Vaccination is good/important	90	96	82	88	NA	NA
HPV vaccination prevents cervical cancer ^a^	7	10	15	22	NA	NA
The HPV vaccine is included in the national immunization programme	3	3	1	4	NA	NA
HPV vaccination prevents genital warts ^b^	1	2	1	1	NA	NA
My doctor recommended the HPV vaccine	0.3	0.6	0	0	NA	NA
**Reasons for refusal of HPV vaccination**						
The HPV vaccine is not recommended for boys	NA	NA	74	78	0	0
Fear of reactions or adverse effects	NA	NA	3	10	51	61
I don’t like/believe in vaccines	NA	NA	0	2	12	18
My daughter/son is too young	NA	NA	4	4	12	14
My daughter/son doesn’t need the HPV vaccine	NA	NA	6	6	8	8
My religion doesn’t approve the HPV vaccine	NA	NA	0	0	6	6
My doctor didn’t recommend the HPV vaccine	NA	NA	2	2	4	4
Other reason(s) not specified	NA	NA	12	12	6	6

NA = Not applicable.

^**a**^25.1% of parents who accepted vaccination of daughters/sons and 37.7% of parents who accepted vaccination of daughters only, knew that the HPV vaccine prevents cancer

^b^ 9.7% of parents who accepted vaccination of daughters/sons and 2.9% of parents who accepted vaccination of daughters only, knew that the HPV vaccine prevents genital warts

The most common reason for refusing HPV vaccination for both children was “fear of reactions or adverse events” reported as the primary reason by 51% of the parents, and as one of them by 61%. Among parents refusing HPV vaccination for sons (but accepting it for daughters), the reason most commonly reported was “the HPV vaccine is not recommended for boys” (74% as the primary reason and 78% as one of them).

### HPV vaccination in the National Immunization Programme (NIP)

Out of 291 parents with a daughter eligible to receive the HPV vaccine through the NIP (9 to 14 years of age),170 (58.4%) reported their daughter had completed the two-dose schedule, and 207 (71.1%) had received at least one dose (Table 4). The most common reason reported for not having a daughter vaccinated or for not having them complete the two-dose regimen was “no vaccination/missed vaccination at school” (51.2% and 75.7% respectively).

**Table 4:** Human papillomavirus (HPV) vaccination coverage among girls 9 to 14 years of age in the National Immunization Programme (NIP) reported by parents (n = 291), Brazil, 2015–2016.

	n	%
Vaccination of daughter(s) against HPV (n = 291)		
Yes (at least one dose) ^a^	207	71.1
No	84	28.9
Reasons for not vaccinating daughter(s) in the NIP (n = 84)		
No vaccination/missed vaccination at school	43	51.2
My daughter is too young	21	25.0
I don’t believe in vaccines/I am against vaccines	16	19.0
My daughter does not need the vaccine	15	17.9
Fear of adverse effects/reactions	8	9.5
My religion does not permit HPV vaccination	1	1.2
My doctor did not recommend the HPV vaccine	1	1.2
Other logistic/access barriers	12	14.3
Reasons for not getting the second dose of HPV vaccine (n = 37)		
No vaccination/missed vaccination at school	28	75.7
Went to a primary health-care center, but could not get vaccinated	7	18.9
I thought that one dose was enough	3	8.1
Other	5	13.5

^a^170of291 received two doses 58.4%.

## Discussion

One year after the inclusion of HPV vaccine in the Brazilian NIP, most parents surveyed were accepting of the HPV vaccination for their daughters (92%) or sons (86%) at the recommended age. Despite the high parental acceptance of HPV vaccine for both daughters and sons, HPV vaccination coverage in Brazil remains only modest for girls (45.1%) and quite poor for boys (16.5%)[6].

Acceptance of the HPV vaccine for girls was also high among parents in Indonesia (96%)[14] and in the US (75%)[15]. In contrast, the level of HPV vaccine acceptance among inner city Caribbean and African American adolescents (44.5%) and their parents (37.5%) was overall lower than what has been reported among other racial/ethnic populations[16]. Parental acceptance of male HPV vaccination in our study population was higher than in France (49%), and comparable to estimates reported in the UK (75%), Germany (72%), and Italy (70%)[17]. Similarly, a nationally representative random sample of 450 Danish parents showed that HPV vaccination of sons was accepted by 80% of respondents[18]. Of note, the survey design of these studies was different from ours in one important aspect: the investigators gave parents information about the main direct benefits of male vaccination, before asking them about their views on HPV vaccination of their sons. Thus, one should be cautious when comparing vaccine acceptance rates from such studies to estimates from those offering parents no information about HPV vaccine prior to the survey. In studies similar to ours, parental acceptance was lower in two surveys in the U.S. (43%[19] and 39%[20]) and comparable to ours in the UK(79%)[21], while in Italy, 71% of parents revealed their intentions to vaccinate their sons against HPV, but only 53.7% reported that their daughters had been vaccinated[22].

HPV vaccine acceptance did not differ substantially by sex of child in two large reviews, although a preference of parents and health care providers to vaccinate females over males was reported in the majority of studies reviewed[23,24]. Nevertheless, many studies included in these reviews were based on the hypothetical availability of an HPV vaccine for boys, and results may not indicate actual acceptance. It was somehow surprising to find high parental acceptance of HPV vaccine for sons in our survey, given that the HPV vaccine was mainly marketed in Brazil as a cancer vaccine for girls, and that parents had limited information about HPV infection in men and its consequences for male health[17,25].

Knowledge about HPV and HPV vaccine was fair in our study population. Although most parents knew about HPV and its association with cervical cancer in women and other cancers in men, this knowledge was not associated with acceptance of HPV vaccination for daughters or sons. Some studies have suggested that parents accept the value of HPV vaccine’s role in cancer prevention[16,26]. In a survey of urban Indian parents, only 27% of men and 24% of women agreed to vaccinate their daughters against HPV; but, after going through an educational fact sheet about cervical cancer and the HPV vaccine, 74% of both men and women were in favor of vaccination[27]. Yet, knowing that HPV causes cervical cancer may not be sufficient for parents to accept HPV vaccination, as most parents rejecting vaccination in our survey were aware of this association. Relatively few parents in our study (10%) cited cancer prevention as one of the reasons to accept HPV vaccination, given that most (86%) knew about the link between HPV and cervical cancer. It is possible that parents perceive cancers occurring later in life as less important on their decision to accept a vaccine given to pre-adolescents/adolescents than other severe diseases, such as meningitis, which might be viewed as a more serious and immediate threat to their children.

In our study, parents who knew that HPV vaccine is more beneficial when given before sexual debut were more likely to accept HPV vaccination (as well as parents who agreed to give their child a vaccine against a STI). Some studies have suggested that parents’ concerns about offering their child a vaccine to prevent sexually transmitted infections and parents’ beliefs that HPV vaccine would promote promiscuity as reasons for not vaccinating[28–30]. There has been controversy regarding the alleged role of HPV vaccine in promoting sexual activity. Concerns among parents about the vaccine’s effect on sexual behavior were reported in a review of barriers to HPV vaccination among US adolescents[31]. Our results suggest that knowledge about HPV sexual transmission did not lead parents to refuse HPV vaccination, but rather made them more likely to accept it. Differences in study populations may account for these diverse findings.

In our survey, parents who knew that HPV vaccine is effective and generally safe were more likely to accept vaccination, as shown in previous studies[15,26,32]. Additionally, trust in vaccines in general and in the NIP were important correlates of parental acceptability. It has been shown that parents in countries with active vaccination policies tended to trust the importance of NIPs, while those in countries with passive vaccination strategies had a greater need for information from health care professionals and public health authorities[17].

General belief in vaccination was the primary reason for parental acceptance of HPV vaccine in our survey. In contrast, a study of a representative sample of parents in the State of California found a much smaller proportion (4.9%) reporting a general belief in recommended vaccinations as one of the reasons for being likely to vaccinate[15]. In our study, parents’ perceptions about vaccines in general were cited more often in their decision to vaccinate than their perceptions about diseases or disease susceptibility. Furthermore, fear of vaccine side effects and distrust in vaccines were the most commonly given reason to refuse HPV vaccination reported in our study. Since parents’ decision to vaccinate children was mainly based on their general belief in vaccines, acceptance rates maydecline if parents are confronted by false arguments against vaccination from anti-vaccination narratives[33].

As far as we know, this is the first population-based survey of HPV vaccine coverage in Brazil since its introduction in the NIP. Our estimates of vaccine coverage for the two-dose course (58%) and for at least one dose (71%) were similar to the ones provided by the NIP, 45% and 72%, respectively[6]. The programme achieved a high coverage (>90%) for the first dose of HPV vaccination early in 2014, when the NIP used a school-based vaccine delivery,but was less than 50% for the second dose later in the same year[34]. It was not clear whether this reduction was caused by an increase in parental vaccine refusal (after the report of clusters of cases of lower limbs paralysis following receipt of HPV vaccine at two schools, interpreted as a mass psychogenic reaction)[7] or it was caused by the change in vaccine delivery from school to health center based strategy, or both[34]. Our results show that parental acceptance of HPV vaccine in Brazil remains high, as does trust of parents in the NIP and its recommended vaccines. School-based approaches to adolescent vaccination implemented in the UK and Australia have achieved high coverage, while approaches based in health care delivery settings tend to be less successful[35]. Moreover, two thirds of parents missing vaccination of their daughter(s) in our study reported either no vaccination at school or other barriers related to vaccine access as the reasons for that. Therefore, only one third of them had actually refused HPV vaccination. Similarly, nearly all parents missing their daughter(s) second-dose of the HPV vaccine reported barriers related to vaccine access at the health care settings as the reason for not completing the vaccination schedule. It is likely that the low coverage of HPV vaccination in Brazil are due to challenges in adolescent vaccine delivery and HPV vaccination barriers at health-care centers, rather than to an increase in parental vaccine refusal.

### Strengths and limitations

Our study had several strengths, including the use of a national sample, and refining our survey instrument extensively through cognitive testing and pretesting. In addition, we did not inform parents about the vaccine’s potential health benefits for females and males before asking them about their views on HPV vaccination (which could have affected vaccine acceptability among study participants). Finally, most studies of attitudes about HPV vaccination come out of high-income countries. We provided much needed research examining HPV vaccination in general and opinions about male vaccination in a middle-income country.

There are many limitations to this study. First, we reported on individual items and have not attempted to combine items in subscales to examine each construct impacted on decision making. Also, one cannot be sure about how the results obtained via a telephone interview might translate into real-life decisions, where medical information and conversation with family will influence parents’ decision process. The response rate (35%) is low and may not seem sufficient to generalize the results to the target population, however, this number is relatively high for a telephone survey. According to the American Association for Public Opinion Research the average telephone response rate in 2015 for the U.S.A was 9.3%[36]. Moreover, refusals in telephone surveys tend to be driven by general unwillingness to participate and non-biased. Thus, it is not likely to have distorted our estimates. In addition, because our estimates of HPV vaccine uptake were based on self-reported data and willingness levels, they may overstate future vaccination behavior, as intent does not always lead to behavior[37]. However, we also provided data on actual vaccination behavior from the subset of parents with daughters in the recommended vaccination age range. Also,more than half of the parent in our sample had children under 9 and so they may have not yet started engaging with thinking about the vaccine. The negative wording of the questions regarding males might have had a negative bias on parent's responses. Lastly, we assumed that parents will be the principal decision makers concerning female and male adolescent vaccination and have not examined adolescents' attitudes about receipt of HPV vaccine. Nonetheless, it may be important to assess how parents and adolescents make decisions about HPV vaccination together.

## Conclusions

Our study shows that most parents in Brazil are interested in vaccinating their daughters and sons against HPV. Nevertheless, HPV vaccination coverage in the NIP remains low. Barriers to access to vaccination in health care settings are likely the main reason for low HPV vaccine uptake, therefore changing back to a school-based vaccine delivery would likely improve vaccine coverage. Regardless of the vaccination strategies adopted, more efforts should be made to educate parents and adolescents about HPV infection and its implications for male and female health[11,38,39]. As for those refusing the vaccine, confidence in vaccines and perception of risk are important, while for those who vaccinate daughters but not sons, understanding about the vaccine as it pertains to males is important. The modifiable factors identified here should be targeted in future interventions to increase HPV vaccine uptake among both males and females in Brazil.

## Supporting information

S1 Dataset (SAV)

S1 Questionnaire Original Questionnaire in Portuguese. (DOCX)

S2 Questionnaire English Questionnaire. (DOCX)
