# Supplementary material for: Low coverage of HPV vaccination in the national immunization programme in Brazil: Parental vaccine refusal or barriers in health-service based vaccine delivery?
Source: PLoS One. 2018 Nov 12;13(11):e0206726. doi: 10.1371/journal.pone.0206726 (PMC6231618; doi:10.1371/journal.pone.0206726)
Supplement: S1 Questionnaire — (DOCX) [file pone.0206726.s002.docx]

**Avaliação da Aceitação Parental da Vacina Contra HPV**

| **Entrevistadora:_________________________** | **Data da Entrevista:_______/______/_____________** |
| --- | --- |
| **Cidade:________________________________** | **Entrevista #:________________________________** |

**SEÇÃO A: INFORMAÇÕES sociodemográficos**

| **A1** | **Quantos anos a senhora tem?** | **\|____\|____\| anos** |
| --- | --- | --- |
| **A2** | **Qual foi a última série ou curso que a senhora concluiu?**  **[REVISAR E CODIFICAR DEPOIS, SE NECESSÁRIO]**  **0** = Nenhum  **1**= Classe de alfabetização / Alfabetização de adultos  **2** = Educação de jovens e adultos  **3** = Ensino fundamental ou 1º grau  **4** = Supletivo ensino fundamental ou 1º grau  **5** = Ensino médio ou 2º grau  **6** = Supletivo ensino médio ou 2º grau  **7** = Curso técnico profissionalizante  **8** = Superior - graduação  **9** = Pós-graduação (Especialização, Mestrado e Doutorado) | **\|____\|____\| série** |
| **A3** | **Qual a sua cor ou raça? [AGUARDARRESPOSTA]**  **1**= Branca  **2**= Preta/Negra  **3** = Parda  **4** = Amarela  **5** = Indígena  **6** = Outra: **\|_____________________________________________________________\|** | **\|_____\|** |
| **A4** | **Qual é sua religião? [AGUARDAR RESPOSTA] [__________________________________________]**  **1** = Católica  **2** = Evangélica - denominação: **\|____________________________________________\|**  **3** = Espírita  **4** = Testemunha de Jeová  **[CONCILIAR LISTA DE denominações e Codificar No final da entrevista]**  **5**= Judaica  **6** = Umbanda  **7** = Candomblé  **8**= Semreligião  **9**= Outra: **\|_____________________________________________________________\|** | **\|_____\|** |
| **A5** | **Qual o seu estado civil? [AGUARDAR RESPOSTA]**  **1** = Casada ou união estável  **2** = Desquitada ou separada  **3** = Divorciada  **4** = Viúva  **5** = Solteira (nunca foi casada / unida) | **\|_____\|** |
| **A6** | **Qual é sua ocupação atual? [AGUARDARRESPOSTA] - - - - - - - - - - - - - - - - - - - - - - - - - - - - - - - - -**  **0** = Nenhum / Sem ocupação / Desempregada  **1**= Dona de casa  **2** = Aposentada  **3** = Estudante  **4** = Profissional liberal / Por conta-própria  **5** = Trabalha (com carteira assinada)  **6** = Outro: \|_________________________________________________________________________\| | **\|_____\|** |
| **A7** | **Você tem algum plano de saúde? - - - - - -- - - - - - - - -- - - - - - - - - - - - - - - - -**{NÃO= **0** ; SIM = **1**} | **\|_____\|** |

**SEÇÃO B: CONHECIMENTO SOBRE O HPV E A VACINA**

**Agora eu vou fazer algumas perguntas sobre o Papiloma Vírus Humano ou HPV. Note que o HPV é diferente do HIV, o vírus que causa a AIDS.**

| **B1** | **A senhora já tinha ouvido falar do Papiloma Vírus Humano, ou HPV?** - - - - - - -{NÃO= **0** ; SIM = **1**}**-** | **\|_____\|** |
| --- | --- | --- |

| ***Mesmo assim, gostaria de saber sua opinião sobre HPV. Eu vou ler algumas afirmações. Por favor, responda se você acha que é ‘Verdadeiro’ ou ‘Falso’.*** | | **Verdadeiro** | **Falso** | **Não Sei** |
| --- | --- | --- | --- | --- |
| **B2** | **O HPV é transmitido pelo uso de banheiro público / piscina / banheira.** |  |  |  |
| **B3** | **O HPV é transmitido por contato sexual.** |  |  |  |
| **B4** | **O HPV é transmitido por via respiratória / pelo ar.** |  |  |  |
| **B5** | **Uma pessoa com HPV geralmente apresenta sintomas.** |  |  |  |
| **B6** | **O HPV é um vírus muito comum.** |  |  |  |
| **B7** | **Homens não podem contrair / pegar HPV.** |  |  |  |
| **B8** | **Mesmo não tendo sintomas, uma pessoa pode transmitir HPV.** |  |  |  |
| **B9** | **O uso de camisinhaprotege completamente contra HPV.** |  |  |  |
| **B10** | **O HPV pode ser curado com antibióticos.** |  |  |  |
| **B11** | **Já existe vacina contra HPV.** |  |  |  |

| **B** | **Sabendo que já existe uma vacina contra HPV, em sua opinião, para que serve essa vacina?**  **[PARA O CASO DE RESPOSTAS VAGAS, EXPLORAR USANDO OS PROBES E CODIFICAR DEPOIS]** | |
| --- | --- | --- |
| **B12** | **Não sei / para prevenir HPV/ para prevenir doença / para ter saúde/ para ficar forte- - - - - - - - - - - - - - - - - -** | **\|____\|** |
| **B13** | **Para prevenir doenças não relacionadas com o HPV**(Câncer de mama, febre, gripe, etc.)**- - -- - - - - - - -- -** | **\|____\|** |
| **B14** | **Para prevenir Câncer relacionado ao HPV**(ex. colo do útero, ânus, pênis) | **\|____\|** |
| **B15** | **Para prevenir DSTnão relacionados** com HPV (ex. gonorreia, herpes, AIDS, etc) | **\|____\|** |
| **B16** | **Para prevenir DSTrelacionados** ao HPV (ex. verrugas genitais / condiloma) | **\|____\|** |
| **B17** | **Outro motivo não especificado: _______________________________________________________________** | **Não \|___\|** |

**SEÇÃO C: ATITUDES SOBRE O HPV E ACEITAÇÃO DA VACINA**

| **C1** | **Você daria a vacina contra HPV para uma filha menor de 18 anos?** - - - - - - - - - - - - - - - - - {NÃO= **0** ; SIM = **1**} | **\|_____\|** |
| --- | --- | --- |

| **[OBS: PARA O CASO DA MÃE RESPONDER “NÂO = 0”]**  **[AGUARDAR RESPOSTA E CODIFICAR]** | | | **[OBS: PARA O CASO DA MÃE RESPONDER “SIM = 1”]**  **[AGUARDAR RESPOSTA E CODIFICAR]** | | |
| --- | --- | --- | --- | --- | --- |
| **C2** | **Por que você não daria a vacina contra HPV? Diga o principal motivo/razão, na sua opinião?**  **1**= Vacinas fazem mal/ não acredito em vacinas;  **2** = Não acredito na vacina contra HPV;  **3** = Minha filha não precisa tomar a vacina contra HPV;  **4** = A vacina é muito cara;  **5** = Medo dos efeitos adversos, reação;  **6** = Minha filha é muita nova para tomar a vacina;  **7**= Meu médico não recomendou;  **8** = Minha religião não permite vacinar contra HPV;  **9**= Outro: _____________________________ | **\|_____\|** | **C4** | **Por que você daria a vacina contra HPV? Diga o principal motivo/razão, na sua opinião?**  **1**= Vacinar é bom / é importante/ acredito em vacinas;  **2** = Está na campanha ou programa nacional;  **3** = A vacina é de graça;  **4** = Todo mundo está dando;  **5** = Meu médico recomendou;  **6** = A vacina previne câncer (geral);  **7** = A vacina previne câncer de colo do útero;  **8** = Previne verrugas genitais/condiloma;  **9** = Outro: _____________________________ | **\|____\|** |
| **C3** | **Tem mais algum motivo para nãodar a vacina? Pense bem. [AGUARDAR, EXPLORAR, CODIFICAR “SIM/NÃO OU 1 a 8”]**  **_______________________________________** | **\|_____\|** | **C5** | **Tem mais algum motivo para dar a vacina? Pense bem. [AGUARDAR, EXPLORAR, CODIFICAR “SIM/NÃO OU 1 a 8”] ____________________________________** | **\|____\|** |

| **C6** | **Você daria a vacina contra HPV para um filho menor de 18 anos?** - - - - - - - - - - - - - - - - - - {NÃO= **0** ; SIM = **1**} | **\|_____\|** |
| --- | --- | --- |

| **[OBS: PARA O CASO DA MÃE RESPONDER “NÂO = 0”]**  **[AGUARDAR RESPOSTA E CODIFICAR]** | | | **[OBS: PARA O CASO DA MÃE RESPONDER “SIM = 1”]**  **[AGUARDAR RESPOSTA E CODIFICAR]** | | |
| --- | --- | --- | --- | --- | --- |
| **C2** | **Por que você não daria a vacina contra HPV? Diga o principal motivo/razão, na sua opinião?**  **1**= Vacinas fazem mal/ não acredito em vacinas;  **2** = Não acredito na vacina contra HPV;  **3** = Meu filho não precisa tomar a vacina contra HPV;  **4** = A vacina é muito cara;  **5** = Medo dos efeitos adversos, reação;  **6** = Meu filho é muita novo para tomar a vacina;  **7** = Meu médico não recomendou;  **8** = Minha religião não permite vacinar contra HPV;  **9** = A vacinanão serve para os meninos;  **10** = Outro: _____________________________  ______________________________________ | **\|_____\|** | **C4** | **Por que você daria a vacina contra HPV? Diga o principal motivo/razão, na sua opinião?**  **1**= Vacinar é bom / é importante/ acredito em vacinas;  **2** = Está na campanha ou programa nacional;  **3** = A vacina é de graça;  **4** = Todo mundo está dando;  **5** = Meu médico recomendou;  **6** = A vacina previne câncer (geral);  **7** = A vacina previne câncer de colo do útero;  **8** = Previne verrugas genitais/condiloma;  **9** = Outro: _____________________________ | **\|____\|** |
| **C3** | **Tem mais algum motivo para nãodar a vacina? Pense bem. [AGUARDAR, EXPLORAR, CODIFICAR “SIM/NÃO OU 1 a 9”]**  **_______________________________________**  **_______________________________________** | **\|_____\|** | **C5** | **Tem mais algum motivo para dar a vacina? Pense bem. [AGUARDAR, EXPLORAR, CODIFICAR “SIM/NÃO OU 1 a 9”]**  **_______________________________________**  **_______________________________________** | **\|____\|** |

***“Eu vou ler algumas afirmações sobre a vacina contra o HPV. Por favor, responda se você ‘Concorda’ ou ‘Não Concorda’ ”*[LER AS OPCÕES DE RESPOSTA AO FIM DE CADA AFIRMAÇÃO]**

|  | | **Vocêconcorda?** | **Vocênãoconcorda?** | **NãoSei** |
| --- | --- | --- | --- | --- |
| C11. | **A vacina contra HPVé eficaz / funciona.** |  |  |  |
| C12. | **Minha filhanão precisa tomar a vacina contra HPV.** |  |  |  |
| C13. | **Meu filhonão precisa tomar a vacina contra HPV.** |  |  |  |
| C14. | **Eu acredito em vacinas de um modo geral.** |  |  |  |
| C15. | **Minha filhatem risco/chance de pegar HPV.** |  |  |  |
| C16. | **Meu filhotem risco/chance de pegar HPV.** |  |  |  |
| C17. | **A vacina contra HPV é muito cara.** |  |  |  |
| C18. | **Eu não daria a vacina contra HPV a minha filhase tivesse que pagar.** |  |  |  |
| C19. | **Eu não daria a vacina contra HPV a meu filhose tivesse que pagar.** |  |  |  |
| C20. | **A vacina contra o HPV não é segura e pode dar reações graves.** |  |  |  |
| C21. | **Meninas de 9 á 13 anos são muito novas para tomar a vacina contra HPV.** |  |  |  |
| C22. | **As reações mais comuns à vacina contra HPV são leves, tipo dor e incômodo no local da injeção.** |  |  |  |
| C23. | **Tomar a vacina contra HPV pode fazer as meninasiniciarem a vida sexual mais cedo.** |  |  |  |
| C24. | **Tomar a vacina contra HPV pode fazer os meninosiniciarem a vida sexual mais cedo.** |  |  |  |
| C25. | **Eu só daria a vacina contra HPV para minha filha se o médico dela recomendasse.** |  |  |  |
| C26. | **Minha religião não permite vacinar contra HPV.** |  |  |  |
| C27. | **Eu confio nas vacinas que são dadas nas campanhas de vacinação** |  |  |  |
| C28. | **Eu só daria a vacina contra HPV a minha filha se minhas amigas também dessem para as filhas delas.** |  |  |  |
| C29. | **Eu não daria uma vacina contra uma doença sexualmente transmissível.** |  |  |  |
| C30. | **Os pais têm obrigação de vacinar seus filhos.** |  |  |  |
| C31. | **A vacina contra HPV não serve para meninos.** |  |  |  |

**SEÇÃO C: ATITUDES SOBRE O HPV E ACEITAÇÃO DA VACINA (CONT.)**

***“Agora, eu vou ler algumas afirmações. Por favor, responda se você acha que é ‘Verdadeiro’, ‘Falso,’ ou ‘Não Sei’ quando não tiver certeza. ”*[LER AS OPCÕES DE RESPOSTA AO FIM DE CADA AFIRMAÇÃO]**

|  | | **Vocêconcorda?** | **Vocênãoconcorda?** | **NãoSei** |
| --- | --- | --- | --- | --- |
| C25. | **A vacina não serve para meninos.** |  |  |  |
| C26. | **Meu filho não precisa da vacina contra HPV.** |  |  |  |
| C28. | **Eu daria a vacina contra HPV a meu filho mesmo tendo de pagar.** |  |  |  |
| C29. | **Tomar a vacina contra o HPV pode fazer as meninos iniciarem a vida sexual mais cedo.** |  |  |  |
| C31. | **Eu daria a vacina para meu filho principalmente para proteger as meninas / mulheres.** |  |  |  |

***“Eu vou ler algumas afirmações sobre a vacina contra o HPV. Por favor, responda se você ‘Concorda’, ‘Não Concorda’ ”*[LER AS OPCÕES DE RESPOSTA AO FIM DE CADA AFIRMAÇÃO]**

|  | | **Verdadeiro** | **Falso** | **Não Sei** |
| --- | --- | --- | --- | --- |
| C32. | **Existe vacina contra o câncer do colo do útero.** |  |  |  |
| C33. | **Não existe vacina contra condiloma e verruga genital.** |  |  |  |
| C34. | **O HPV pode causar câncer do colo do útero.** |  |  |  |
| C35. | **O câncer de colo do útero não é uma causa comum de morte por câncer entre as mulheres** |  |  |  |
| C36. | **O HPV não causa câncer em homens.** |  |  |  |
| C37. | **O HPV pode causar verrugas genitais / condiloma / “crista de galo.”** |  |  |  |
| C38. | **Se o exame preventivo / Papanicolau é normal, então a mulher não tem HPV.** |  |  |  |
| C39. | **Meninas que tomaram a vacina contra o HPV não precisam mais fazer o exame preventivo / Papanicolau.** |  |  |  |
| C40. | **A vacina contra o HPV funciona melhor quando dada antes do início da vida sexual.** |  |  |  |

**SEÇÃO D: PRÀTICAS SOBRE O HPV E A VACINA**

***“Agora vamos falar um pouco sobre sua saúde.***

***“Agora vamos falar um pouco sobre sua saúde.***

| **D1** | **Você já fez o exame preventivo ou Papanicolau alguma vez na vida - - - - - - - - - - - - - - - -**{NÃO= **0** ; SIM = **1**}  **[SE FOR NÃO, SALTE PARA D3]** | **\|_____\|** |
| --- | --- | --- |
| **D2** | **Nos últimos 3 anos, você fez algum exame preventivo ou Papanicolau? - - - - -- - -** { NÃO= **0** ; SIM = **1** } | **\|_____\|** |
| **D3** | **Alguma vez um médico lhe informou que a senhora teve câncer de colo do útero?**{ NÃO= **0** ; SIM = **1** } | **\|_____\|** |
| **D4** | **Você tem alguma pessoa querida na família ou não, que morreu de câncer? - -- - -**{ NÃO= **0** ; SIM = **1** } | **\|_____\|** |
| **D5** | **Você já tomou algum reforço da vacina tríplice contra a difteria/tétano/coqueluche?**  {NÃO**=0;** SIM=**1**; NÃO SEI=**2}**  **[OBS:SE ELA RESPONDER QUE SIM]** | **\|_____\|** |
| **D6** | **Foi nos últimos 10 anos? - - - - - - - - - - - - - - - - - - -** { NÃO**= 0 ;** SIM =**1**; NÃO SEI =**2 }** | **\|_____\|** |
| **D7** | **Você já tomou a vacina contra Hepatite B? - - - - - - - - - - - - - - - - - -** { NÃO**= 0 ;** SIM =**1**; NÃO SEI =**2 }** | **\|_____\|** |
| **D8** | **Se a vacina contra HPV funcionasse em qualquer idade, você tomaria? - - - - - - - -** { NÃO= **0** ; SIM = **1** } | **\|_____\|** |
| **D9** | **Você conhece outras mães que deram a vacina contra o HPV aos seus filhos?**  **[LER AS OPCÕES DE RESPOSTA]**  **2** = Nunca perguntei / conversei com alguém sobre isso.  **1** = Sim, conheço.  **0** = Não conheço. | **\|_____\|** |

**SEÇÃO E: Programa Nacional de Imunizações**

| **E1** | **Você tem alguma filha de 10 a 14 anos de idade? - - - - - - - - - - - - - - - - - - - - - - - - - - - - - -**{NÃO= **0** ; SIM = **1**}  **[SE ELA RESPONDER QUE “NÃO”, SALTAR PARA A CONCLUSÃO]** | **\|_____\|** |
| --- | --- | --- |
| **E2** | **Sua filha(s) estuda(m) na escola pública ou particular?**  **1=** PÚBLICA;  **2**= PARTICULAR;  **3**=NÃO ESTUDA | **\|_____\|** |
| **E3** | **Você ouviu falar sobre a campanha de vacinação contra o HPV nas escolas este ano?-**{ NÃO= **0** ; SIM = **1** }  **[SE ELA RESPONDER QUE “NÃO”, SALTAR PARA E20.]** | **\|_____\|** |

| **“Você ficou sabendo da campanha…” [LER TODAS AS OPÇÕES PAUSADAMENTE]** | | **Sim** | **Não** |
| --- | --- | --- | --- |
| E5. | **…pela escola?”** |  |  |
| E6. | **…pelos amigos ou familiares?** |  |  |
| E7. | **…pela TV?”** |  |  |
| E8. | **…pela Internet?”** |  |  |
| E9. | **...pelo jornal?”** |  |  |
| E10. | **...pelo rádio?”** |  |  |

| **“Você recebeu algum material sobre a campanha da vacina contra o HPV como…”**  **[LER TODAS AS OPÇÕES PAUSADAMENTE]** | | **Sim** | **Não** |
| --- | --- | --- | --- |
| E13. | **…um folheto/panfleto informativo?”** |  |  |
| E14. | **…um termo de recusa / consentimento da vacinação contra o HPV?”** |  |  |
| E15. | **…um cartão de vacina?”** |  |  |
| E16. | **…uma caderneta do adolescente?”** |  |  |

| **E19** | **Você ficou satisfeita com as informaçõessobre a vacina contra HPV durante a campanha?**  **[LER TODAS AS OPÇÕES SEGUIDAMENTE]**  { NÃO**= 0;** MAIS OU MENOS =**1**; SIM =**2}** | **\|_____\|** |
| --- | --- | --- |
| **E20** | **Sua(s) filha(s) foi(foram) vacinada(s) contra HPV na campanha de 2014?**  {NÃO**=0;**SIM=**1**; NÃO SEI=**2}** | **\|_____\|** |

| **[OBS: PARA O CASO DA MÃE RESPONDER “NÂO = 0”]** | | | **[OBS: PARA O CASO DA MÃE RESPONDER “SIM = 1”]** | | |
| --- | --- | --- | --- | --- | --- |
| **E21** | **Por que ela não recebeu a vacina da campanha?**  **[AGUARDAR RESPOSTA E CODIFICAR]**  **1**= Estou preocupada com os efeitos adversos;  **2** = Minha religião não permite vacinar contra o HPV;  **3** = Meu médico não recomendou;  **4** = Ela é jovem demais;  **5** = Ela faltou o dia de vacinação na escola / Não foi ao posto;  **6** = Minha filha está gravida.  **7** =Não foi oferecida na escola dela;  **8**= Eu não soube da campanha.  **9**= Outro = \|__________________________\| | **\|____\|** | **E22** | **Quantas doses da vacina ela recebeu na campanha desteano?**  **[SE ELA RESPONDER“1 = UMA DOSE”]**  **[SE ELA RESPONDER**  **“1 = UMA DOSE”]**  **1** = Uma dose  **2**= Duas doses  **3** = Não sei | **\|_____\|** |
|  |  |  | **E23** | **Porque sua filha não recebeu a segunda dose da vacinaesse ano?**  **[AGUARDAR RESPOSTA E CODIFICAR]**  **1**= Ela já tinha recebido uma dosefora da campanha.  **2** =Ela teve um evento/reação adverso com a primeira dose.  **3**=Eu achava que só precisava tomar uma dose  **4** =Ela faltou o dia de vacinação na escola.  **5** =Eu não fui ao posto de saúde. / Não foi oferecida na escola.  **6** =Fui ao posto, mas ela não pode receber.  (Não tinha vacina, a fila estava grande, o posto estava fechado)  **7** =Minha filha está gravida.  **8** =O médico contraindicou.  **9** =Não sei  **10** =Outro: \|________________________\| | **\|_____\|** |

**CONCLUSÃO**

***“Obrigado por sua colaboração!”***

***“Obrigado pelasuaparticipação!”***
