# Supplementary material for: Low coverage of HPV vaccination in the national immunization programme in Brazil: Parental vaccine refusal or barriers in health-service based vaccine delivery?
Source: PLoS One. 2018 Nov 12;13(11):e0206726. doi: 10.1371/journal.pone.0206726 (PMC6231618; doi:10.1371/journal.pone.0206726)
Supplement: S2 Questionnaire — (DOCX) [file pone.0206726.s003.docx]

ENGLIGH TRANSLATION

**EVALUATION OF PARENTAL ACCEPTANCE OF THE HPV VACCINE**

Date: ___________________________

Call Log Number: ___________________________

*Here, record the corresponding ID for the number dialed.*

Obtain consent to proceed with the interview. If the parent agrees to participate, begin the questionnaire.

City: 1_ Belém

2_ Belo Horizonte

3_ Brasília

4_ Porto Alegre

5_ Rio de Janeiro

6_ Salvador

7_ São Paulo

[SECTION A – SOCIODEMOGRAPHIC INFORMATION OF INTERVIEWEE]

**A1 How old are you?**

[WAIT FOR RESPONSE]

**___________________________**

**A2 What is your religious affiliation?**

[WAIT FOR RESPONSE]

1_ Catholic

2_ Evangelical / Protestant

3_ Spiritist

4_ Jehovah’s Witness

5_ Jewish

6_ Umbanda

7_ Candomblé

8_ No religion

9_ Other

**A2.1 What? _________________________________________________**

[WAIT FOR RESPONSE]

**A3 What is your marital status?**

[WAIT FOR RESPONSE]

1_ Married or Civil Union

2_ Separated

3_ Divorced

4_ Widowed

5_ Single (never married)

**A4 What is your current occupation?**

[WAIT FOR RESPONSE]

1_ None / Unemployed

2_ Homemaker

3_ Retired

4_ Student

5_ Self-employed

6_ Working

7_ Public Servant

8_ Other

**A4.1 What? _________________________________________________**

[WAIT FOR RESPONSE]

**A5 Do you have health insurance?**

[WAIT FOR RESPONSE]

1_ Yes

0_ No

**A6 What is your race/ethnicity?**

[WAIT FOR RESPONSE]

1_ White

2_ Black

3_ Mixed

4_ Asian

5_ Indigenous

6_ Other

**A6.1 What? _________________________________________________**

[WAIT FOR RESPONSE]

**A7 What was the last educational level you completed?**

[WAIT FOR RESPONSE]

1_ No formal education

2_ Adult literacy class

3_Adult School

4_ Primary school

5_ Middle school

6_ High school

7_ High school equivalence (GED)

8_ Technical certificate

9_ College degree (bachelors)

10_ Graduate degree (masters, doctorate)

**A8 Sex/gender:**

1_ Female

2_ Male

[SECTION B – KNOWLEDGE ABOUT HPV AND THE VACCINE]

**B1 Have you heard of HUMAN PAPILLOMAVIRUS or HPV?**

1_Yes

0_No

Either way, I would like to hear your opinion about HPV. I will read some statements. Please, respond “YES” or “NO.”

[READ THE ITEMS ONE AT A TIME]
[READ RESPONSE OPTIONS “YES” OR “NO”]

[WAIT FOR RESPONSE]

**B2 HPV is transmitted through the use of public bathrooms /pools/ showers.**

1_YES or [READ]

0_NO [READ]

2_I don’t know [DO NOT READ]

**B3 HPV is transmitted by sexual contact.**

1_YES or [READ]

0_NO [READ]

2_I don’t know [DO NOT READ]

**B4 HPV is spread by airborne transmission.**

1_YES or [READ]

0_NO [READ]

2_I don’t know [DO NOT READ]

**B5 Someone with HPV usually has symptoms.**

1_YES or [READ]

0_NO [READ]

2_I don’t know [DO NOT READ]

**B6 HPV is a very common virus.**

1_YES or [READ]

0_NO [READ]

2_I don’t know [DO NOT READ]

**B7 Men cannot catch HPV.**

1_YES or [READ]

0_NO [READ]

2_I don’t know [DO NOT READ]

**B8 Even without symptoms, someone can transmit HPV.**

1_YES or [READ]

0_NO [READ]

2_I don’t know [DO NOT READ]

**B9 Condom use fully protects against HPV.**

1_YES or [READ]

0_NO [READ]

2_I don’t know [DO NOT READ]

**B10 HPV can be cured with antibiotics.**

1_YES or [READ]

0_NO [READ]

2_I don’t know [DO NOT READ]

**B11 A vaccine against HPV already exists.**

1_YES or [READ]

0_NO [READ]

2_I don’t know [DO NOT READ]

**B12 Knowing that a vaccine against HPV exists, in your opinion, what is the vaccine for?**

[CAN ACCEPT MORE THAN ONE RESPONSE]

[WAIT FOR RESPONSE – IN CASE OF VAGUE RESPONSES, TRY USING THE PROBES AND CODE AFTERWARDS]

[PROBES:]

[WHAT DO YOU MEAN TO SAY WITH…?]

[WHAT TYPE OF DISEASE?]

[WHAT TYPE OF CANCER?]

[WHAT TYPE OF SEXUALLY TRANSMITTED DISEASE?]

B12.1_I don’t know / to prevent HPV / to prevent disease / to be healthy / to stay strong

B12.2_ To prevent diseases not related to HPV (ex. breast cancer, fever, flu, etc.)

B12.3_ To prevent cancers related to HPV (ex. cervix, anus, penis)

B12.4_ To prevent STDs not related to HPV (ex. gonorrhea, herpes, AIDS, etc.)

B12.5_ To prevent STDs related to HPV (ex. genital warts / condyloma)

B12.6_ Other reason not specified?

B12.7 What? _________________________________________________

[SECTION C – ATTITUDES ABOUT HPV AND VACCINE ACCEPTANCE]

**C1 Would you give the HPV vaccine to a DAUGHTER less than 18 years?**

[WAIT FOR RESPONSE]

1_Yes [GO TO C4]

0_No

**C2 [If NO] Why would you not give the HPV vaccine? State the main reason, in your opinion.**

[WAIT ANDMARK ONLY ONE RESPONSE]

1_ Fear of adverse effects/reaction, vaccines are bad

2_ My daughter is too young to receive the vaccine

3_ My daughter doesn’t need the HPV vaccine

4_ I don’t believe in vaccines / I don’t like vaccines

5_ I don’t believe in / don’t like the HPV vaccine

6_ The vaccine is too expensive

7_ My doctor didn’t recommend the HPV vaccine

8_ My religion doesn’t approve the HPV vaccine

9_ Other reason not specified

C2.1 What? _________________________________________________

**C3 Is there another reason to not give the vaccine?**

1_Yes

0_No [GO TO C6]

**C3.1 What?**

[WAIT ANDMARK ONLY ONE RESPONSE]

1_ Fear of adverse effects/reaction, vaccines are bad

2_ My daughter is too young to receive the vaccine

3_ My daughter doesn’t need the HPV vaccine

4_ I don’t believe in vaccines / I don’t like vaccines

5_ I don’t believe in / don’t like the HPV vaccine

6_ The vaccine is too expensive

7_ My doctor didn’t recommend the HPV vaccine

8_ My religion doesn’t approve the HPV vaccine

9_ Other reason not specified

C3.1.1 What? _________________________________________________

**C4 [IF YES] Why would you give the HPV vaccine? State the main reason, in your opinion.**

[WAIT ANDMARK ONLY ONE RESPONSE]

1_ To prevent HPV / Vaccination is good / Vaccination is important / I believe in vaccines

2_ The HPV vaccine is included in the national immunization program

3_ The vaccine is free

4_ Everyone else is giving it

5_ My doctor recommended the HPV vaccine

6_ HPV vaccination prevents cancer [general]

7_ HPV vaccination prevents cervical cancer

8_ HPV vaccination prevents genital warts / condyloma

9_ Other reason not specified

C4.1 What? _________________________________________________

**C5 Is there another reason to give the vaccine?**

1_Yes

0_No

**C5.1 What?**

[WAIT ANDMARK ONLY ONE RESPONSE]

1_ To prevent HPV / Vaccination is good / Vaccination is important / I believe in vaccines

2_ The HPV vaccine is included in the national immunization program

3_ The vaccine is free

4_ Everyone else is giving it

5_ My doctor recommended the HPV vaccine

6_ HPV vaccination prevents cancer [general]

7_ HPV vaccination prevents cervical cancer

8_ HPV vaccination prevents genital warts / condyloma

9_ Other reason not specified

C5.1.1 What? _________________________________________________

**C6 Would you give the HPV vaccine to a SON less than 18 years?**

[WAIT FOR RESPONSE]

0_Yes [GO TO C9]

1_No

**C7 [IF NO] Why would you not give the HPV vaccine? State the main reason, in your opinion.**

[WAIT ANDMARK ONLY ONE RESPONSE]

1_ The HPV vaccine is not for boys

2_ Fear of adverse effects/reaction, vaccines are bad

3_ My son is too young to receive the vaccine

4_ My son doesn’t need the HPV vaccine

5_ I don’t believe in vaccines / I don’t like vaccines

6_ I don’t believe in / don’t like the HPV vaccine

7_ The vaccine is too expensive

8_ My doctor didn’t recommend the HPV vaccine

9_ My religion doesn’t approve the HPV vaccine

10_ Other reason not specified

C7.1 What? _________________________________________________

**C8 Is there another reason to not give the vaccine?**

1_Yes

0_No

**C8.1 What?**

[WAIT ANDMARK ONLY ONE RESPONSE]

1_ The HPV vaccine is not for boys

2_ Fear of adverse effects/reaction, vaccines are bad

3_ My son is too young to receive the vaccine

4_ My son doesn’t need the HPV vaccine

5_ I don’t believe in vaccines / I don’t like vaccines

6_ I don’t believe in / don’t like the HPV vaccine

7_ The vaccine is too expensive

8_ My doctor didn’t recommend the HPV vaccine

9_ My religion doesn’t approve the HPV vaccine

10_ Other reason not specified

C8.1.1 What? _________________________________________________

**C9 [IF YES] Why would you give the HPV vaccine? State the main reason, in your opinion.**

[WAIT ANDMARK ONLY ONE RESPONSE]

1_ To prevent HPV / Vaccination is good / Vaccination is important / I believe in vaccines

2_ The HPV vaccine is included in the national immunization program

3_ The vaccine is free

4_ Everyone else is giving it

5_ My doctor recommended the HPV vaccine

6_ HPV vaccination prevents cancer [general]

7_ HPV vaccination prevents penile cancer

8_ HPV vaccination prevents genital warts / condyloma

9_ Other reason not specified

C9.1 What? _________________________________________________

**C10 Is there another reason to give the vaccine?**

1_Yes

0_No [GO TO C11]

**C10.1 What?**

[WAIT ANDMARK ONLY ONE RESPONSE]

1_ To prevent HPV / Vaccination is good / Vaccination is important / I believe in vaccines

2_ The HPV vaccine is included in the national immunization program

3_ The vaccine is free

4_ Everyone else is giving it

5_ My doctor recommended the HPV vaccine

6_ HPV vaccination prevents cancer [general]

7_ HPV vaccination prevents penile cancer

8_ HPV vaccination prevents genital warts / condyloma

9_ Other reason not specified

C10.1.1 What? _________________________________________________

I will read some statements about the HPV vaccine. Please, respond “YES, I AGREE” or “NO, I DISAGREE.”

[READ RESPONSE OPTIONS AT THE END OF EACH STATEMENT]

**C11 The HPV vaccine is efficacious / it works.**

1_YES or [READ]

0_NO [READ]

2_I don’t know [DO NOT READ]

**C12 I think that my DAUGHTER does not need the HPV vaccine.**

1_YES or [READ]

0_NO [READ]

2_I don’t know [DO NOT READ]

**C13 I think that my SON does not need the HPV vaccine.**

1_YES or [READ]

0_NO [READ]

2_I don’t know [DO NOT READ]

**C14 I generally believe in vaccines.**

1_YES or [READ]

0_NO [READ]

2_I don’t know [DO NOT READ]

**C15 I think that my DAUGHTER is at risk / has a chance of getting HPV.**

1_YES or [READ]

0_NO [READ]

2_I don’t know [DO NOT READ]

**C16 I think that my SON is at risk / has a chance of getting HPV.**

1_YES or [READ]

0_NO [READ]

2_I don’t know [DO NOT READ]

**C17 I think that the HPV vaccine is very expensive.**

1_YES or [READ]

0_NO [READ]

2_I don’t know [DO NOT READ]

**C18 I would not give the HPV vaccine to My DAUGHTER if I had to pay.**

1_YES or [READ]

0_NO [READ]

2_I don’t know [DO NOT READ]

**C19 I would not give the HPV vaccine to my SON if I had to pay.**

1_YES or [READ]

0_NO [READ]

2_I don’t know [DO NOT READ]

**C20 I think the HPV is not safe / I think it can cause severe reactions.**

1_YES or [READ]

0_NO [READ]

2_I don’t know [DO NOT READ]

**C21 GIRLS between 9 and 13 years are too young to get the HPV vaccine.**

1_YES or [READ]

0_NO [READ]

2_I don’t know [DO NOT READ]

**C22 The most common reactions from the HPV vaccine are minor, such as pain and discomfort at the injection site.**

1_YES or [READ]

0_NO [READ]

2_I don’t know [DO NOT READ]

**C23 You think getting the HPV vaccine cause GIRLS to become sexually active much earlier.**

1_YES or [READ]

0_NO [READ]

2_I don’t know [DO NOT READ]

**C24 You think getting the HPV vaccine cause BOYS to become sexually active much earlier.**

1_YES or [READ]

0_NO [READ]

2_I don’t know [DO NOT READ]

**C25 You would only give the HPV vaccine to your DAUGHTER if her doctor recommended it.**

1_YES or [READ]

0_NO [READ]

2_I don’t know [DO NOT READ]

**C26 Your religion does not allow the HPV vaccine.**

1_YES or [READ]

0_NO [READ]

2_I don’t know [DO NOT READ]

**C27 You trust the national immunisation program.**

1_YES or [READ]

0_NO [READ]

2_I don’t know [DO NOT READ]

**C28 You would only give your DAUGHTER the HPV vaccine if your friends also gave it to their daughters.**

1_YES or [READ]

0_NO [READ]

2_I don’t know [DO NOT READ]

**C29 You would not give your child a vaccine against a sexually transmitted infection.**

1_YES or [READ]

0_NO [READ]

2_I don’t know [DO NOT READ]

**C30 Parents have a responsibility to vaccinate their children.**

1_YES or [READ]

0_NO [READ]

2_I don’t know [DO NOT READ]

**C31 The HPV vaccine is not for BOYS.**

1_YES or [READ]

0_NO [READ]

2_I don’t know [DO NOT READ]

**C32 There is a vaccine against cervical cancer.**

1_YES or [READ]

0_NO [READ]

2_I don’t know [DO NOT READ]

**C33 There is no vaccine against genital warts.**

1_YES or [READ]

0_NO [READ]

2_I don’t know [DO NOT READ]

**C34 HPV can cause cervical cancer.**

1_YES or [READ]

0_NO [READ]

2_I don’t know [DO NOT READ]

**C35 Cervical cancer is NOTa common cause of cancer death among women.**

1_YES or [READ]

0_NO [READ]

2_I don’t know [DO NOT READ]

**C36 HPV does not cause cancer in men.**

1_YES or [READ]

0_NO [READ]

2_I don’t know [DO NOT READ]

**C37 HPV can cause genital warts / condyloma.**

1_YES or [READ]

0_NO [READ]

2_I don’t know [DO NOT READ]

**C38 If a preventive exam/Pap smear is normal, then a woman does not have HPV.**

1_YES or [READ]

0_NO [READ]

2_I don’t know [DO NOT READ]

**C39 GIRLS that receive the HPV vaccine do not need to have preventive exams.**

1_YES or [READ]

0_NO [READ]

2_I don’t know [DO NOT READ]

**C40 The HPV vaccine works better when it is given before the start of sexual activity.**

1_YES or [READ]

0_NO [READ]

2_I don’t know [DO NOT READ]

[SECTION D: PRACTICES AROUND HPV AND THE VACCINE]

NOW, WE ARE GOING TO TALK A LITTLE BIT ABOUT YOUR HEALTH.

**D1 Have you had a cervical cancer screening or Paptest at least once before?**

[WAIT FOR RESPONSE]

1_Yes

0_No

**D2 Have you had a cervical cancer screening or Pap smearin the last 3 years?**

[WAIT FOR RESPONSE]

1_Yes

0_No

**D3 Has a doctor ever told you that you had cervical cancer?**

[WAIT FOR RESPONSE]

1_Yes

0_No

**D4 Has someone in your family died of cancer?**

[WAIT FOR RESPONSE]

1_Yes

0_No

**D4.1 Who?**

[CAN ACCEPT MORE THAN ONE RESPONSE]

D4.1.1_ Father / Mother

D4.1.2_ Brother / Sister

D4.1.3_ Son / Daughter

D4.1.4_ Grandfather / Grandmother

D4.1.5_ Great-Grandfather / Great-Grandmother

D4.1.6_ Uncle / Aunt

D4.1.7_ Cousin

**D5 Have you had the Diphtheria, Pertussis, and Tetanus (DPT) vaccine?**

[WAIT FOR RESPONSE]

1_Yes [DO NOT READ]

0_No [DO NOT READ]

2_I don’t remember / I don’t know [DO NOT READ]

**D5.1 Was it in the last 10 years?**

[WAIT FOR RESPONSE]

1_Yes [DO NOT READ]

0_No[DO NOT READ]

2_I don’t remember / I don’t know [DO NOT READ]

**D6 Have you had the Hepatitis B vaccine?**

[WAIT FOR RESPONSE]

1_Yes [DO NOT READ]

0_No [DO NOT READ]

2_I don’t remember / I don’t know [DO NOT READ]

**D7 If the HPV vaccine worked for any age, would you get it?**

[WAIT FOR RESPONSE]

1_Yes

0_No

**D8 Do you know other parents who had their children vaccinated with HPV vaccine?**

[WAIT FOR RESPONSE]

1_Yes

0_No

[SECTION E: NATIONAL IMMUNIZATION PROGRAM]

**E1 Do you have a DAUGHTER between 9 and 14 years old?**

[WAIT FOR RESPONSE]

1_Yes

0_No

**E2 Does your daughter study in a public or private school?**

[WAIT FOR RESPONSE]

1_ Public

2_ Private

3_ Not in school

**E3 Have you heard about the HPV vaccine campaign for GIRLS?**

[WAIT FOR RESPONSE]

1_Yes

0_No

**E4 How did you hear about the campaign?**

[READ EACH RESPONSE ONE AT A TIME]

[CAN ACCEPT MORE THAN ONE RESPONSE]

E4.1_Through school?

E4.2_ Through friends or colleagues?

E4.3_ ThroughTV?

E4.4_Through the internet?

E4.5_Through the newspaper?

E4.6_ Through radio?

**E5 Did you receive any materials about the HPV vaccination campaign, like…?**

[READ EACH RESPONSE ONE AT A TIME]

[CAN ACCEPT MORE THAN ONE RESPONSE]

E5.1_ Informative pamphlet?

E5.2_ Consent / Refusal form for HPV vaccine?

E5.3_ Vaccine report

E5.4_ Adolescent booklet?

E5.5_ Nothing

**E6 Were you satisfied with the information about the HPV vaccine during the campaign?**

[WAIT FOR RESPONSE]

1_Yes

0_No

2_ Somewhat

**E7 Was your DAUGHTER vaccinated against HPV in the campaign that happened in the last year (2014)?**

[WAIT FOR RESPONSE]

1_ Yes

0_ No

2_ I don’t know

**E7.1 Why did she not receive the vaccine in the campaign?**

[CAN ACCEPT MORE THAN ONE RESPONSE]

E7.1.1_ I am afraid of adverse effects.

E7.1.2_ My religion does not permit HPV vaccination.

E7.1.3_My doctor did not recommend the HPV vaccine.

E7.1.4_ My daughter is too young.

E7.1.5_ My daughter missed the vaccination day at school. / There was no vaccination at the health post.

E7.1.6_ My daughter is pregnant.

E7.1.7_ It was not offered at her school.

E7.1.8_ I did not know about the campaign.

E7.1.9_ Other reason not specified.

E7.1.1 What? _________________________________________________

[ASK AGAIN]**Any other reason?**

**E8 How many doses of the HPV vaccine has she received to date?**

[WAIT FOR RESPONSE]

1_ One

2_ Two

3_ Three

(Minimum of 1 and Maximum of 3 doses)

**E9 [IF ONE DOSE ONLY] Why did your DAUGHTER not receive a second vaccine dose?**

[CAN ACCEPT MORE THAN ONE RESPONSE]

E9.1_ It was not offered at her school.

E9.2_ She had an adverse event / reaction with the first dose.

E9.3_ I thought she only needed to take one dose.

E9.4_ My daughter missed the vaccination day at school.

E9.5_ She went to the health post, but she was not vaccinated. (They did not have the vaccine, the line was long, the health post was closed)

E9.6_ My daughter is pregnant.

E9.7_ She had already received a dose outside of the campaign.

E9.8_ I don’t know

E9.9_ Other reason not specified

E9.1 What? _________________________________________________

**E10 Do you have ANOTHER DAUGHTER between 9 and 13 years old that should have received the first dose of the HPV vaccine in this year’s campaign (2015)?**

[WAIT FOR RESPONSE AND CODE]

1_Yes

0_No

**E11 Did she receive the vaccine?**

[WAIT FOR RESPONSE AND CODE]

1_Yes

0_No

**E11.1 Why did she not receive the vaccine in the campaign?**

[CAN ACCEPT MORE THAN ONE RESPONSE]

[WAIT FOR RESPONSE]

E11.1.1_ I am afraid of adverse effects.

E11.1.2_ My religion does not permit HPV vaccination.

E11.1.3_ My doctor did not recommend the HPV vaccine.

E11.1.4_ My daughter is too young.

E11.1.5_ My daughter missed the vaccination day at school. / There was no vaccination at the health post.

E11.1.6_ My daughter is pregnant.

E11.1.7_ It was not offered at her school.

E11.1.8_ I did not know about the campaign.

E11.1.9_ Other reason not specified.

**E11.9 What? _________________________________________________**

THANK YOU VERY MUCH FOR YOUR PARTICIPATION! [READ]

YOU MAY RECEIVE A CALL FROM MY SUPERVISOR TO CHECK THE QUALITY OF MY WORK. [READ]
